# Neoadjuvant therapy versus upfront surgery for potentially resectable pancreatic cancer: A Markov decision analysis

**DOI:** 10.1371/journal.pone.0212805

**Published:** 2019-02-28

**Authors:** Alison Bradley, Robert Van Der Meer

**Affiliations:** 1 Department of Management Science, Strathclyde Business School, University of Strathclyde, Glasgow, Scotland, United Kingdom; 2 West of Scotland Pancreatic Cancer Unit, Glasgow Royal Infirmary, Glasgow, Scotland, United Kingdom; University of Texas MD Anderson Cancer Center, UNITED STATES

## Abstract

**Background:**

Neoadjuvant therapy has emerged as an alternative treatment strategy for potentially resectable pancreatic cancer. In the absence of large randomized controlled trials offering a direct comparison, this study aims to use Markov decision analysis to compare efficacy of traditional surgery first (SF) and neoadjuvant treatment (NAT) pathways for potentially resectable pancreatic cancer.

**Methods:**

An advanced Markov decision analysis model was constructed to compare SF and NAT pathways for potentially resectable pancreatic cancer. Transition probabilities were calculated from randomized control and Phase II/III trials after comprehensive literature search. Utility outcomes were measured in overall and quality-adjusted life months (QALMs) on an intention-to-treat basis as the primary outcome. Markov cohort analysis of treatment received was the secondary outcome. Model uncertainties were tested with one and two-way deterministic and probabilistic Monte Carlo sensitivity analysis.

**Results:**

SF gave 23.72 months (18.51 QALMs) *versus* 20.22 months (16.26 QALMs). Markov Cohort Analysis showed that where all treatment modalities were received NAT gave 35.05 months (29.87 QALMs) *versus* 30.96 months (24.86QALMs) for R0 resection and 34.08 months (29.87 QALMs) *versus* 25.85 months (20.72 QALMs) for R1 resection. One-way deterministic sensitivity analysis showed that NAT was superior if the resection rate was greater than 51.04% or below 75.68% in SF pathway. Two-way sensitivity analysis showed that pathway superiority depended on obtaining multimodal treatment in either pathway.

**Conclusion:**

Whilst NAT is a viable alternative to traditional SF approach, superior pathway selection depends on the individual patient’s likelihood of receiving multimodal treatment in either pathway.

## Introduction

Outcomes for pancreatic cancer remain poor despite advances in surgical technique and adjuvant treatment [1,2]. Early complete surgical resection is the only potentially curative treatment, with surgery followed by adjuvant therapy becoming the standard of care for resectable pancreatic cancer [3]. Up to 50% of patients fail to receive adjuvant therapy due to: post-operative complications, early metastases nullifying the potential benefits of high-risk surgery, and reduced performance status [4,5]. Neoadjuvant therapy (NAT) has emerged as an alternative to traditional surgery-first (SF) approach. Postulated benefits include: avoiding futile surgery by identifying aggressive tumour types, eliminating micrometastesis, multimodal treatment completion and increased R0 resection rates [6,7].

There is a lack of randomized controlled trials (RCTs) comparing SF and NAT [8]. Despite promising results from cohort studies and phase II trials, meta-analysis have reported only marginal benefit of NAT in terms of overall and disease-free survival [7,9–12]. Critics have therefore highlighted the limitations of drawing optimistic conclusions from small studies that are underpowered [6,7]. Two previous Markov decision-analysis found marginal benefit with NAT for resectable only cases [9,13]. Only one previous Markov decision analysis compared efficacy of both pathways for potentially resectable disease and found no conclusively superior pathway [14].

The aim of this study is to synthesize best current evidence within a Markov decision-analysis model comparing SF and NAT pathways for the management of potentially resectable pancreatic cancer on an intention-to-treat basis. This study aims to improve on previous iterations by performing Markov cohort analysis assessing impact of treatment received on survival outcomes.

## Materials and methods

### Markov model

**Fig 1:**
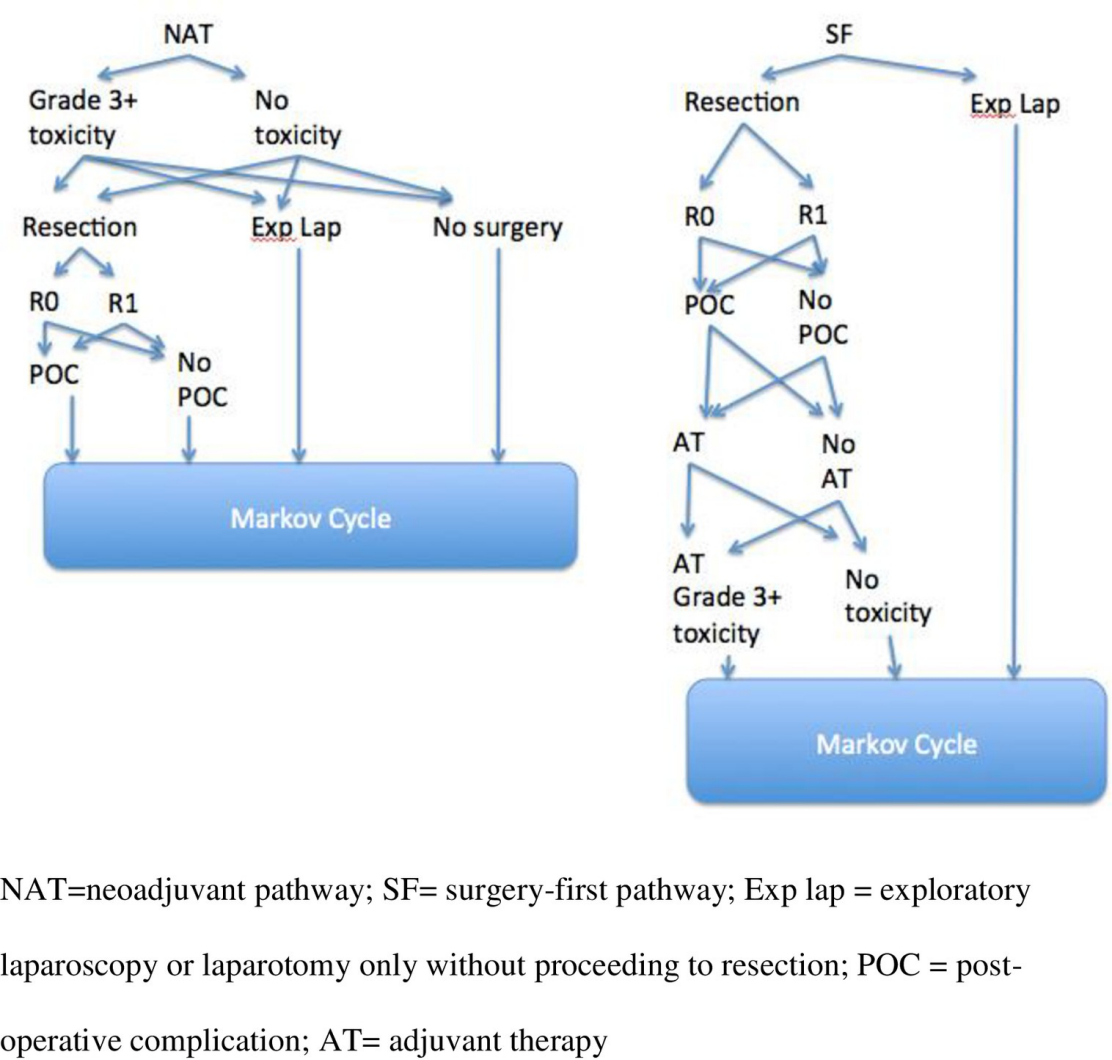
Overview of the structure of the Markov decision-tree.

TreeAge Pro 2017 (TreeAge Software Ins., Williamstown, MA) was used to construct a Markov cohort decision analysis model in an advanced decision-tree format comparing base case, surgery first followed by adjuvant therapy (which included chemotherapy, chemoradiotherapy, or both), to NAT (which included chemotherapy and/or chemoradiotherapy) followed by re-staging and, if possible, surgical resection (Fig 1). Upon completion of treatment, cohorts entered the Markov health-state transition model with possible survival states including: alive without disease, alive with disease and dead. Each Markov cycles equated to 1 month with maximum follow-up of 60-cycles or until death.

### Outcome measures

Cumulative payoffs were calculated in life months and quality-adjusted-life-months (QALMs), which scaled survival from 0 (equivalent to death) to 1 [9,13] based on indicies taken from published literature [15,16] and World Health Organization and European Quality of Life Survey [17–19].

### Data sources and transition probabilities

Source data was identified through comprehensive literature search of MEDLINE, Embase, PubMed and Cochrane database and Cochrane database of Clinical Trials following the PRISMA checklist [20] (S1 Fig). For each of the searches, the entire database was included from the year 2000 up to and including 31^st^ October 2018, with no further date restrictions or limits applied. Following screening, reference lists and citations of all included papers were manually searched to identify any additional articles until no new articles were identified. The lead reviewer performed search design and data extraction with the second author performing independent quality assurance. Discrepancies were resolved by inter-reviewer discussion. The following data was extracted from each study: study details (country, year, design, number, mean age, sex, co-morbidity profile and presenting disease stage of participants), details of treatment protocols, treatment outcomes (treatment completion rates, rates of tumour resection, R0 resection rates, drug toxicity data, post-operative complication rates, overall survival and disease-free survival) and risk of bias data.

The inclusion criteria was RCTs and prospective phase II and III studies of NAT for treatment of pancreatic cancer, published in English language since 2000, involving chemo/radiotherapy-naive human subjects over 18 years of age with preoperatively staged pancreatic cancer as potentially resectable. Included trials had to report: protocol design, number of participants per arm, median age and co-morbidities of subjects, pre-treatment staging of pancreatic cancer, toxicity profile, results of post neoadjuvant re-staging, resection rates, post-operative complications defined by Clavien-Dindo system, and survival data. Retrospective and cohort studies, case series and case reports were excluded as were studies from identical patient cohorts and trials involving intra-operative radiotherapy and trials including disease other than pancreatic cancer.

As the majority of trials were single arm, to populate the SF pathway the same databases were searched for RCTs of surgery and adjuvant therapy, with the same inclusion and data reporting criteria (S2 Fig). The outcomes of this group could introduce biased because by definition these patients have survived surgery and not developed early metastatic disease and also had to have adequate performance status to be randomized to adjuvant therapy even if they did not receive adjuvant therapy. To overcome this issue cohort studies comparing NAT and SF, with the otherwise same inclusion criteria and data reporting requirements, were also included in the SF arm and solely used to offer comparison across outcomes of resection, R0 resection rates and receipt of adjuvant therapy (S3 Fig).

The Cochrane Collaboration’s risk of bias tool [21] and ROBINS-I tool (Risk Of Bias In Non-randomized Studies—of Interventions) [22] were used to assess the quality and risk of bias of each included trial (S1 and S2 Tables and S4 Fig). Furthermore the potential impact of bias and uncertainty on all variables within the model were extensively tested through probabilistic and deterministic sensitivity analysis.

### Statistical analysis

Markov model transition probabilities were based on weighted pooled estimates of proportions from included studies, calculated using Freeman-Tukey arcsine square root transformation under random effects model to account for heterogeneity [23]. Survival time was based on time from diagnosis. Gillen et al. approach to calculating weighted median survival time was used as evidence has shown that weighted averaging of medians cannot achieve unbiased pooled estimates of survival time [24,25]. This approach is based on averaging parameter estimates of a presumed density function of survival. The pooled distribution parameter is used to recalculate the estimate of the median from the pooled distribution parameter [24]. In this case the pooled distribution parameter is the exponential distribution which implies a time constant hazard rate corresponding to the sole distribution parameter λ. From this the weighted estimate of median survival (*m*_*p*_) is derived from the formula [24]:

$$mp=(\sum_{i=1}^{k}\frac{wi}{mi})-1$$

where *m*_*i*_ is median survival within the study population *i* (with *i* being 1 to *k* where *k* is the number of included studies) [24]. *w*_*i*_ is the study specific weight function derived from number of study participants divided by total number of evaluable patients [24].

### Sensitivity analysis

Model uncertainties for all included components were tested with one and two-way deterministic sensitivity analysis with baseline transition probabilities for each variable altered between highest and lowest reported values. Probabilistic Monte Carlo sensitivity analysis was set to 10000 cycles with model probabilities sampled from the data distribution of each variable. Data for each variable was fitted against 55 possible distributions with best fit determined by the Anderson Darling statistic.

## Results

50 phase II/III studies met the inclusion criteria and were included in the NAT arm of the model, 4 of which were randomized. 9 of these studies offered comparison with upfront surgery. For the SF pathway 15 studies were RCTs, 10 of which offered comparison between adjuvant regimes, 5 of which offered comparison between adjuvant therapy and surgery only. 16 cohort studies were also included in the SF pathway to offer comparison across outcomes of resection rates, R0 resection, and rates of receiving adjuvant therapy (Table 1). Probability estimates and ranges and quality of life utilities are displayed in Table 2.

**Table 1:** Summary of included trials.

Reference	Type of Study	Treatment Regime	N =	Disease Free Survival in months	Overall Survival in months
Al-Sukhun et al. [26]	Prospective Phase II Trial	CRT + surgery	20		13.4
Cardenes et al.[27]	Prospective Phase II Trial	CRT + surgery	28		10.3
Casadei et al. [28]	Prospective Phase II Trial	CRT + surgery	18		28.3
Cetin et al. [29]	Prospective Phase II Trial	CRT + surgery	11		
Chakraborty et al. [30]	Prospective Phase II Trial	CRT + surgery	13	2.4	9.1
Crane et al. [31]	Prospective Phase II Trial	CRT + surgery	69		19.2
Epelbaum et al. [32]	Prospective Phase II Trial	CRT + surgery	20		8
Esnaola et al. [33]	Prospective Phase II Trial	CRT + surgery	37	10.4	11.8
Evans et al. [34]	Prospective Phase II Trial	CRT + surgery	86	15.4	22.7
Fiore et al. [35]	Prospective Phase II Trial	CRT + surgery	34	20	19.2
Golcher et al [36]	Prospective Phase II Trial	CRT + surgery	121		
Golcher et al. [37]	Prospective Phase II Trial	CRT + surgery	33	8.4	17.4
Heinrich et al. [38]	Prospective Phase II Trial	CT + surgery	28	9.2	26.5
Herman et al. [39]	Prospective Phase II Trial	CRT + surgery	49	7.8	13.9
Hong et al. [40]	Prospective Phase II Trial	CRT + surgery	50	10.4	17.3
Jang et al. [41]	Prospective Phase II Trial	CRT + surgery	27		21
Jensen et al. [42]	Prospective Phase II Trial	CRT + surgery	23		11.5
Joensuu et al. [43]	Prospective Phase II Trial	CRT + surgery	33	18	25
Kim et al. [44]	Prospective Phase II Trial	CRT + surgery	68		18.2
Landry et al. [45]	Prospective Phase II Trial	CRT + surgery	21	14.2	19.4
Laurent et al. [46]	Prospective Phase II Trial	CRT + surgery	22	8	17
Le Scodan et al. [47]	Prospective Phase II Trial	CRT + surgery	41		9.4
Lee et al. [48]	Prospective Phase II Trial	CT + surgery	43	10	16.6
Leone et al. [49]	Prospective Phase II Trial	CRT + surgery	39	10.2	16.7
Lin et al. [50]	Prospective Phase II Trial	CRT + surgery	42		10.3
Lind et al. [51]	Prospective Phase II Trial	CRT + surgery	17		19
Magnin et al. [52]	Prospective Phase II Trial	CRT + surgery	32		16
Magnino et al. [53]	Prospective Phase II Trial	CRT + surgery	23		14
Marti et al. [54]	Prospective Phase II Trial	CRT + surgery	26	7	13
Mattiucci et al. [55]	Prospective Phase II Trial	CRT + surgery	40		15.5
Massucco et al. [56]	Prospective Phase II Trial	CRT + surgery	28	10	15.4
Maximous et al. [57]	Prospective Phase II Trial	CRT + surgery	25		12
Mornex et al. [58]	Prospective Phase II Trial	CRT + surgery	41		9.4
Motoi et al. [59]	Prospective Phase II Trial	CT+ surgery	35		19.7
Moutardier et al. [60]	Prospective Phase II Trial	CRT + surgery	19		20
O’Reilly et al.[61]	Prospective Phase II Trial	CT + surgery	38		27.2
Palmer et al. [62]	Prospective Phase II Trial	CT + surgery	50		13.6
Pipas et al. [63]	Prospective Phase II Trial	CRT + surgery	37		17.3
Pister et al. [64]	Prospective Phase II Trial	CRT + surgery	37		12
Sahora et al.[65]	Prospective Phase II Trial	CT + surgery	25		16
Satoi et al.[66]	Prospective Phase II Trial	CRT + surgery	35		24.5
Sherman et al. [67]	Prospective Phase II Trial	CRT + surgery v CT + surgery	45	34	29/42
Small et al. [68]	Prospective Phase II Trial	CRT + surgery	29	9.9	11.8
Talamonti et al. [69]	Prospective Phase II Trial	CRT + surgery	20		
Tinchon et al. [70]	Prospective Phase II Trial	CT + surgery	12		
Turrini et al. [71]	Prospective Phase II Trial	CRT + surgery	34		15.5
Van Buren et al. [72]	Prospective Phase II Trial	CRT + surgery	59	6.6	16.8
Varadhachary et al. [73]	Prospective Phase II Trial	CRT + surgery	90	13.2	17.4
Vento et al. [74]	Prospective Phase II Trial	CRT + surgery	22		30.2
Wilkowski et al. [75]	Prospective Phase II Trial	CRT + surgery	93	5.6	9.3
Regine et al.[76]	RCT	Surgery + CRT	230		17.1
Neoptolemos et al. [77]	RCT	Surgery +CT	551	14.1	23
VanLaethem et al.[78]	RCT	Surgery +CRT	45	11.8	24.3
Schmidt et al.[79]	RCT	Surgery +CRT	53	15.2	26.5
Reni et al.[80]	RCT	Surgery +CRT	51	11.7	26.2
Yoshitomi et al.[81]	RCT	Surgery +CT	49	12	29.8
Shimoda et al.[82]	RCT	Surgery +CT	29	14.6	21.5
Uesaka et al.[83]	RCT	Surgery +CT	187	22.9	46.5
Neoptolemos et al.[84]	RCT	Surgery +CRT	145	10.7	15.9
Ueno et al.[85]	RCT	Surgery +CT	58	11.4	22.3
Oettle et al.[86]	RCT	Surgery +CT	179	13.4	22.8
Kosuge et al.[87]	RCT	Surgery +CT	45	8.6	12.5
Smeenk et al. [88]	RCT	Surgery +CRT	110	18	21.6
Morak et al.[89]	RCT	Surgery +CR	59	12	19
Neoptolemo et al.[90]	RCT	Surgery + CT	366		25.5
Regine et al.[76]	RCT	Surgery +CRT	221		20.5
Neoptolemos et al.[77]	RCT	Surgery +CT	537	14.3	23.6
VanLaethem et al.[78]	RCT	Surgery +CT	45	10.9	24.4
Schmidt et al.[79]	RCT	Surgery +CT	57	11.5	28.5
Reni et al.[80]	RCT	Surgery + CT	49	15.2	31.6
Yoshitomi et al.[81]	RCT	Surgery + CT	50	2.3	21.2
Shimoda et al.[82]	RCT	Surgery +CT	28	10.5	18
Uesaka et al.[83]	RCT	Surgery + CT	190	11.3	25.5
Neoptolemos et al.[84]	RCT	Surgery +CT	147	15.3	20.1
Ueno et al. [85]	RCT	Surgery Only	60	5	18.4
Oettle et al.[86]	RCT	Surgery Only	175	6.7	20.2
Kosuge et al.[87]	RCT	Surgery Only	44	10.2	15.8
Smeenk et al.[88]	RCT	Surgery Only	108	14.4	19.2
Morak et al.[89]	RCT	Surgery Only	61	7	18
Neoptolemo et al.[90]	RCT	Surgery + CT	364		28
Al-Sukhun et al.[26]	Prospective Phase II Trial	Surgery + adjuvant therapy	21		18.1
Casadei et al.[28]	Prospective Phase II Trial	Surgery + adjuvant therapy	20		27.5
Golcher et al. [36]	Prospective Phase II Trial	Surgery + adjuvant therapy	58		21
Golcher et al.[37]	Prospective Phase II Trial	Surgery + adjuvant therapy	33	8.7	14.4
Lind et al.[51]	Prospective Phase II Trial	Surgery + adjuvant therapy	35		11
Massucco et al.[56]	Prospective Phase II Trial	Surgery + adjuvant therapy	44	8	14
Satoi et al.[66]	Prospective Phase II Trial	Surgery + adjuvant therapy	41		18.5
Vento et al.[74]	Prospective Phase II Trial	Surgery + adjuvant therapy	25		35.9
Jang et al.[41]	Prospective Phase II Trial	Surgery + adjuvant therapy	23		12
DeGus et al.[91]	Retrospective Cohort	Surgery + adjuvant therapy	6840		24.2
Mellon et al.[92]	Retrospective Cohort	Surgery + adjuvant therapy	241		22.1
Nurmi et al.[93]	Retrospective Cohort	Surgery + adjuvant therapy	150	13	26
Shubert et al.[94]	Retrospective Cohort	Surgery + adjuvant therapy	216		13
Artinya et al.[95]	Retrospective Cohort	Surgery + adjuvant therapy	419		19
Ielpo et al.[96]	Prospective Cohort	Surgery + adjuvant therapy	36		22.1
Roland et al.[97]	Prospective Cohort	Surgery + adjuvant therapy	85		
DeGus et al. [98]	Retrospective Cohort	Surgery + adjuvant therapy	11316		Resectable: 24.5 Borderline: 20.0 Locally advanced: 15.5
Mokdad et al.[99]	Retrospective Cohort	Surgery + adjuvant therapy	6015		21
Chen et al.[100]	Retrospective Cohort	Surgery + adjuvant therapy	98		17
Tzeng et al.[101]	Prospective Cohort	Surgery + adjuvant therapy	52		25.3
Fujii et al.[102]	Prospective Cohort	Surgery + adjuvant therapy	71		13.1
Fujii et al.[103]	Prospective Cohort	Surgery + adjuvant therapy	416		Resectable: 23.5 Borderline: 20.1
Papalezova et al.[104]	Retrospective Cohort	Surgery + adjuvant therapy	92		13
Hirono et al.[105]	Prospective Cohort	Surgery + adjuvant therapy	124		13.7
Murakami et al.[106]	Retrospective Cohort	Surgery + adjuvant therapy	25		11.6

CRT = chemoradiotherapy CT = chemotherapy

**Table 2:** Summary of transition probabilities, parameters of data distribution and payoff utilities for quality adjusted life months (QALMs).

Variable	Baseline Transition Probability (95% CI)	Range	Standard Deviation	Variance	Data Distribution: parameters (Anderson Darling Statistic)	Study Reference
Grade 3+ toxicity with NAT	0.35 (0.28–0.43)	0–1.0	0.03799	0.00144	Generalized Extreme Value: k = 0.45856 σ = 0.01111 μ = 0.00904 (0.55904)	[26–35,37–44,46–55,57–64,67–72,75]
Resection in NAT pathway	0.41 (0.33–0.49)	0–0.86	0.00848	7.1972E-5	Generalized Extreme Value: k = 0.15727 σ = 0.00545 μ = 0.00618 (0.36129)	[26,27,29–37,41–52,54–61,63–66,68–69,71,73–75]
Exploratory Laparoscopy/Laparotomy	0.1 (0.07–0.13)	0–0.36	0.00349	1.2182E-5	Generalized Pareto: k = 0.06879 σ = 0.00306 μ = -5.1223E-4 (1.3525)	[26–29,32–38,40–45,47–49,51,52,54,56–69,71–75]
R0 resection NAT pathway	0.29 (0.21–0.36)	0–0.74	0.0068	4.6303E-5	Johnson SB: γ = 1.7195 δ = 1.0417 λ = 0.04849 ξ = -0.00113 (0.35896)	[27–33,35–39,41,43–46,48–51,53–56,59,61,63,65,66,71,74,75]
Grade 3–4 post-operative complication NAT pathway	0.35 (0.19–0.53)	0.11–0.64	0.02702	7.3021E-4	Generalized Extreme Value: k = -0.45505 σ = 0.03128 μ = 0.04101 (0.1996)	[28,36,37,41,56,74]
Grade 5 post-operative complication NAT pathway	0.02 (0.01–0.03)	0–0.36	0.00097	9.4387E-7	Pareto 2: α = 0.34207 β = 1.3899E-13 (-13.983)	[28,31,34,36–38,40,44,47,48,51,54,56–61,64,65,67–70,72–74]
Resection SF pathway	0.94 (0.90–0.96)	0.70–1.0	0.1219	0.01486	Burr: k = 0.0595 α = 10.327 β = 0.00112 (0.12818)	[26,28,36,37,41,51,56,66,74,91–97,99–106]
R0 resection SF pathway	0.56 (0.51–0.62)	0.16–0.86	0.09869	0.00974	Pearson 5: α = 0.61636 β = 7.0460E-4 (0.18259)	[26,28,36,37,41,51,66,74,91–94,96,97,99,101–106]
Grade 3–4 post-operative complication SF pathway	0.22 (0.13–0.33)	0.04–0.54	0.01297	0.0002	Log-Pearson 3: α = 66.845 β = -0.09425 γ = 2.0838 (0.29235)	[28,36,37,41,51,56,74,96,97,101,102]
Grade 5 post-operative complication SF pathway	0.07(0.02–0.13)	0–0.36	0.00948	8.9795E-5	Cauchy: σ = 0.00373 μ = 0.00639 (0.38658)	[28,36,37,41,51,56,74,96,106]
Receiving adjuvant therapy	0.61(0.57–0.66)	0.26–0.94	0.10088	0.01018	Burr: k = 0.26048 α = 2.145 β = 9.2071E-4 (0.18949)	[41, 77, 79,92,94,96–99,101,102,104–106]
Adjuvant toxicity grade 3+	0.43(0.25–0.62)	0.09–0.98	0.02753	0.00076	Log-Pearson 3: α = 1916.0 β = -0.02672 γ = 47.081 (0.34508)	[76,77,79,81,85,86,88,90]
**Survival State**	**Utility for QALM**
Living with stable pancreatic cancer	0.81
Undergoing chemo/radiotherapy	0.81
Experiencing chemo/radiotherapy complications	0.53
Recovering from pancreatic surgery	0.59
Experiencing surgical complications	0.48
Living with unresectable disease and pre-operative quality-of-life	0.65

### Results of Markov decision-analysis

Intention-to-treat analysis of the treatment pathways, based on baseline transition probabilities, showed that SF pathway gave 23.72 months (18.51 QALMs) compared to 20.22 months (16.26 QALMs) for NAT pathway. The results of Markov cohort analysis are outlined in Table 3 and demonstrated superiority of the NAT pathway for patients who received all treatment modalities.

**Table 3:** Results from Markov cohort analysis.

	NAT Pathway	SF Pathway
**R0 Resection**	35.05 months (29.87 QALMs; POC = 29.76 QALMs)	Received Adjuvant Therapy: 30.96 months (24.86 QALMs; POC = 24.75 QALMs; AT = 21.82 QALMs; POC and AT = 21.71 QALMs) No Adjuvant Therapy: 24.03 months (20.12 QALMs; POC = 20.01QALMs)
**R1 Resection**	34.08 months (29.87 QALMs; POC = 29.76 QALMs)	Received Adjuvant Therapy: 25.85 months (20.72 QALMs; POC = 20.61 QALMs; AT = 18.20 QALMs; POC and AT = 18.09 QALMs) No Adjuvant Therapy: 21.26 months (17.56 QALMs; POC = 17.45 QALMs)
**Exploratory Laparoscopy or Laparotomy**	10.86 months (7.22 QALMs)	10.48 months (6.97 QALMs)
**No Surgery**	10.86 months (7.06 QALMs)	

POC = post-operative complication grade 3 or 4; AT = adjuvant therapy resulting in grade 3 or 4 toxicity

### Deterministic sensitivity analysis

Deterministic sensitivity analysis tested the sensitivity of the results of the model to variations in parameters of specific model variables by altering the parameters between highest and lowest reported values. One-way deterministic sensitivity analysis determined the effect on the overall results of the model by varying the parameter of each variable individually. Two-way deterministic sensitivity analysis determined the effect on the model of altering the parameters of two variables simultaneous.

**Fig 2:**
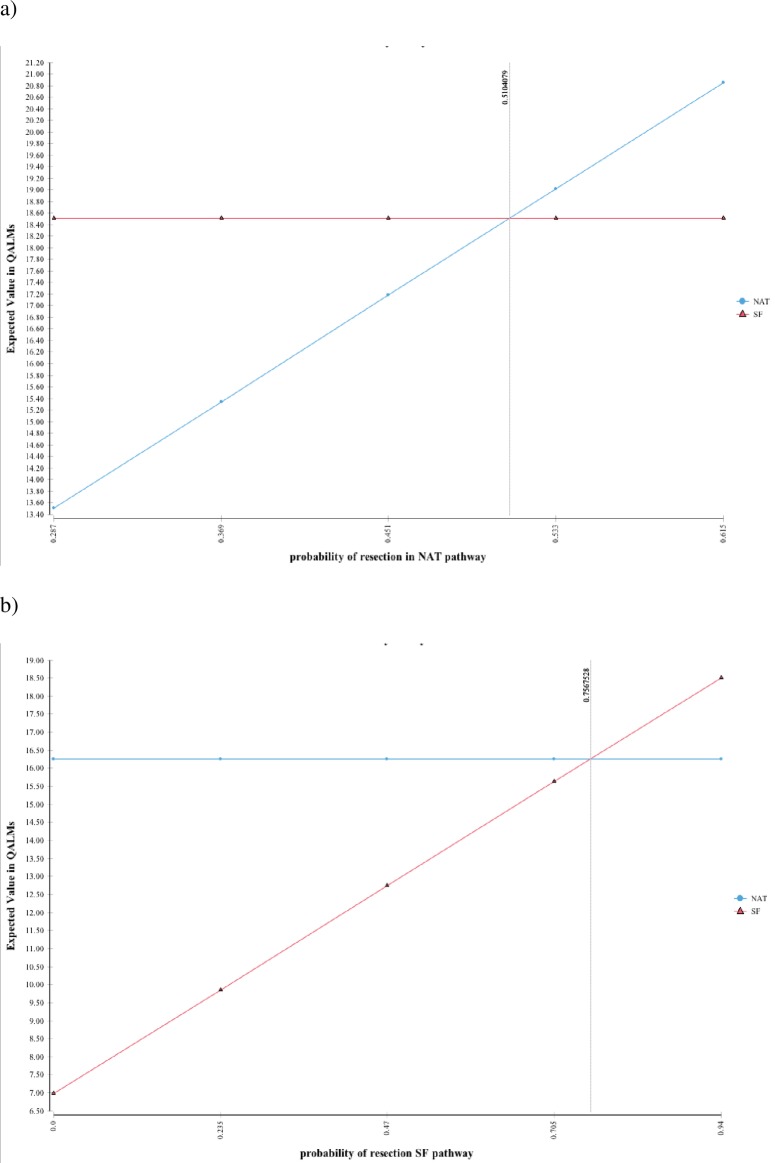
One-way deterministic sensitivity analysis. This figure shows the effect of altering the baseline probability of resection first in the NAT pathway then in the SF pathway on overall model outcome.

One-way deterministic sensitivity analysis showed that NAT was the superior treatment pathway if the probability of achieving resection in the NAT pathway was greater than 51.04% or the probability of achieving resection in the SF pathway was less than 75.68% (Fig 2).

**Fig 3:**
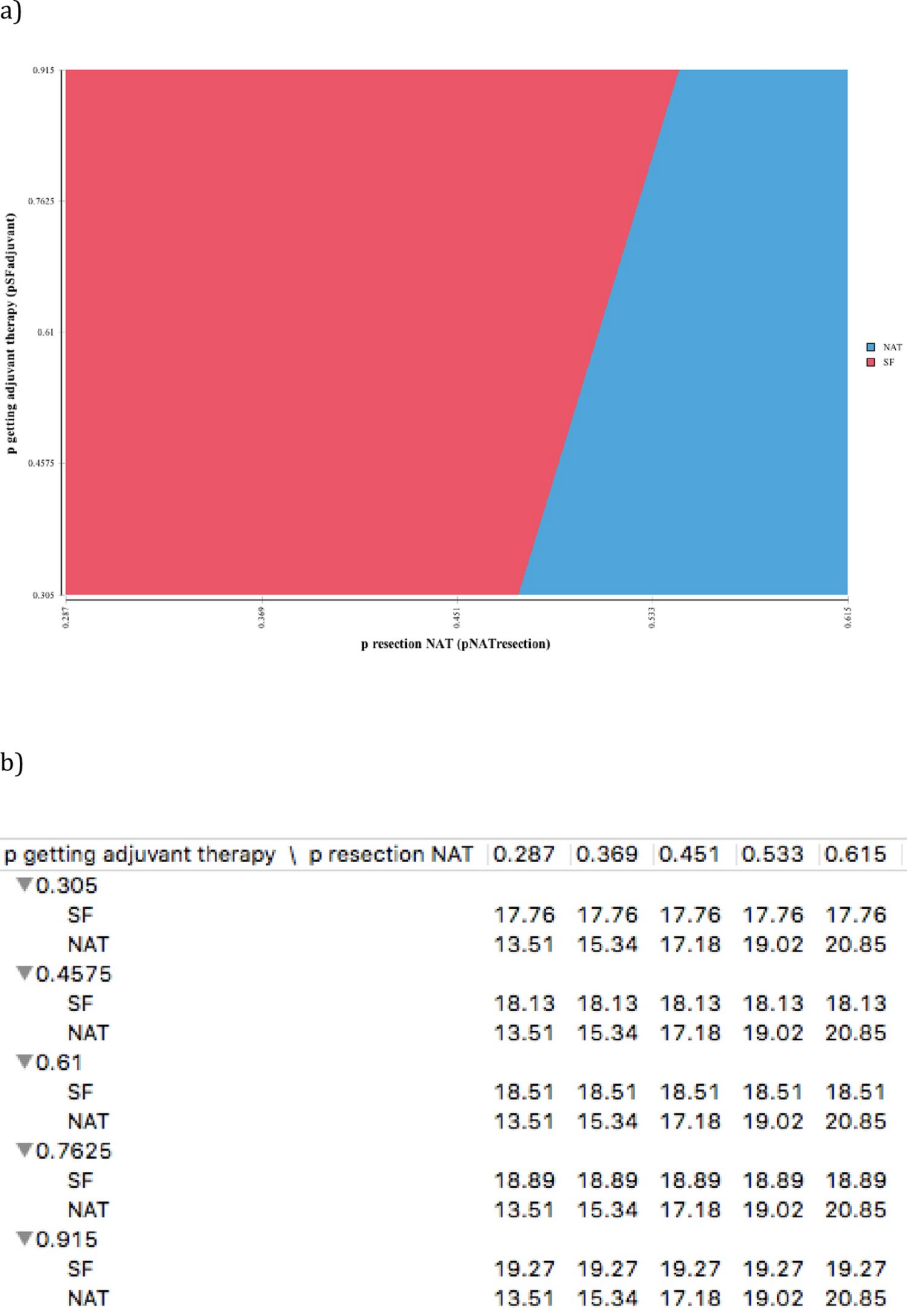
Two-way sensitivity analysis. Y-axis shows probability of receiving adjuvant therapy in SF pathway and x-axis shows probability of receiving resection in NAT pathway. The red area represents where patients, given competing probability of receiving multimodal treatment in competing pathways, would benefit from SF approach. The blue area represents where NAT would be the superior treatment option in terms of quality-adjusted survival. The corresponding predicted survival time in QALMs are detailed below. X and Y-axis provide altering probabilities of multimodal treatment in each pathway with corresponding survival time given in QALMs.

Two-way deterministic sensitivity analysis demonstrated that treatment superiority depended on receiving multimodal treatment (resection in NAT pathways and adjuvant therapy in SF pathway). Fig 3A shows the thresholds at which competing pathways offer superior outcomes with Fig 3B providing corresponding probability thresholds and predicted resulting quality-adjusted survival time.

### Probabilistic sensitivity analysis

Probabilistic sensitivity analysis tested the level of confidence in the model output in relation to uncertainty in model input by determining the distribution of the input data for each variable from the median, standard deviation and variance of the input data. The input data was then fitted against 55 possible data distributions with the best fit determined by the Anderson Darling statistic (Table 2). All possible parameter values for each variable within the model were therefore tested by drawing probabilities from the data distribution when probabilistic Monte Carlo sensitivity analysis was set to simulate 10000 patients cycling through the model.

The results of probabilistic Monte Carlo sensitivity analysis showed that SF gave a mean survival time of 19.72 months (range 5.57–22.95) compared to 17.16 months (range 16.50–17.38) for NAT with standard deviation 2.68 and 0.19, and variance 7.17 and 0.04 in SF and NAT pathways respectively. When minimum significant difference was set at 3.65 months or greater, the model reported indifference in superior pathway selection frequency.

## Discussion

The role of NAT in treatment of pancreatic cancer is an ongoing area of debate [107]. Markov decision analysis is a powerful tool offering analysis of complex medical decisions therefore this study provides important interim analysis and contributes to only three existing studies that utilize evidence-based Markov decision-analysis to compare NAT and SF pathways [9,13,14]. Our analysis showed that SF pathway gave an additional 3.5 months (2.25 QALMs) but neither pathway was conclusively superior. Markov cohort analysis of outcomes where multimodal treatment was received in both pathways (resection in NAT and adjuvant therapy in SF) demonstrated superior outcomes with NAT pathway. One-way sensitivity analysis showed that NAT was superior on in intention-to-treat basis if the probability of resection in the NAT pathway was above 51.04%. Base-case probability of resection in NAT pathway was 41%. This could represent NAT pathway allowing time to filter out more aggressive tumours, hence avoiding futile, expensive and high-risk surgery [6,7]. Conversely critics of NAT would argue that this indicates loosing the window of resectability [6,7]. Critics of NAT have also raised concerns that it could increase post-operative complications, a claim not substantiated in our analysis, which is also corroborated by previous studies [9,14].

Three Markov Decision-Analysis studies comparing NAT and SF pathways for pancreatic cancer exist [9,13,14]. One of these studies most closely resembled this study by focusing on potentially resectable pancreatic cancer, therefore including borderline and locally advanced cases in the NAT pathway to capture the effect of conversion to resectability on overall pathway analysis [9]. As with our findings it did not demonstrate on overall conclusively superior pathway on an intention-to-treat basis (NAT 18.6 months versus 17.1 months) [14]. Two other Markov decision analysis studies have reported superiority of NAT pathway but they focus on resectable only cases and based their model on literature from a single search engine [13,14]. One study, based on phase I/II trials, reported 22 months for NAT *versus* 20 months for SF [9]. De Gus *et al*. demonstrated larger survival gain with NAT (32.2 versus 26.7 months) but their analysis mostly included retrospective studies [13]. Preliminary results from the PREOPANC-1 trial, a multicenter phase III RCT comparing NAT and SF for borderline resectable PC, have reported improved survival with NAT on an intention-to-treat basis (17.1 months versus 13.5 months) [108]. Although PREOPANC-1 is a very different study to the one presented here in terms of design and statistical methodology, the results do echo our findings in reporting that higher reported resection rates in the SF pathway do not equate with superior overall survival time for patients treated in a SF pathway [108]. Furthermore the subgroup analysis of resected cases in the PREOPANC-1 trial reported superior survival time with NAT (29.9months versus 16.6months), which further corroborates the results our Markov cohort analysis [108].

### Strengths and limitations

Improving upon previous iterations, this study, based on a comprehensive search of multiple search engines, went beyond intention-to-treat base-case analysis to perform Markov cohort analysis of treatment received, which provided important results favoring NAT. These findings corroborate those of prospective and retrospective experiences of NAT, which have demonstrated favorable survival outcomes ranging from 26 to 45 months [13] and findings reported by Abbott *et al*. who, although performing Markov analysis for cost-effectiveness, reported similar outcomes [109].

Considering at base-case 39% in SF pathway did not received adjuvant therapy and 59% in NAT pathway did not undergo resection, this adds an important dimension to the ongoing debate. Two-way sensitivity analysis demonstrated that the superior treatment pathway depended on an individual’s probability of receiving multimodal treatment in either pathway. This highlights the need to work towards personalized predictive medicine to assist in the selection of the most appropriate treatment pathway at individual patient level.

Like existing Markov decision analysis based on data from published studies, this study also shares the limitations of the existing body of evidence: heterogeneity, lack of randomization, potential bias, small and underpowered studies [6,7]. Furthermore definitions of radiological and surgical resectability, R0 resection and staging protocol can vary across trials further compounding the issue of heterogeneity of synthesized data [8]. Such heterogeneity could account for why, at base-case analysis, the probability of R0 resection in the NAT pathway was smaller than anticipated particularly when considering that the PREOPANC-1 trial has reported higher rates of R0 resection (65%) in the NAT arm of their trial [8,108]. Although the uncertainty of this variable was extensively tested through sensitivity analysis and found not to affect the overall model outcomes, this highlights the potential impact of heterogeneity of data on model output. To address the issue of heterogeneity, this study based transition probabilities on weighted pooled proportion estimates calculated using Freeman-Tukey arcsine square root transformation under random effects model [23]. Furthermore probabilistic Monte Carlo sensitivity analysis sampled model probabilities from the entire range of the data distribution and provided assessment of the extent of variance and standard deviation within the model. Unlike previous Markov decision-analysis, weighted survival times were based on the Gillen *et al*. formulae as evidence has shown that unbiased pooled estimates of median survival times cannot be achieved by weighted averaging of medians [24,25]. Quality adjusted survival time is limited by the lack of studies measuring quality-of-life across the treatment trajectory for pancreatic cancer. This study utilized best available data that was shared with existing decision-analysis studies, which enhanced comparability. Our model did not assume return to full health after an intervention or event but accounted for the impact of therapy, surgery, complications and disease reoccurrence when calculating quality-adjusted survival. For both survival and quality-adjusted survival, uncertainty was rigorously tested across every variable in the model through probabilistic and deterministic sensitivity analysis.

## Conclusion

In the absence of large multicenter RCTs, this study utilizes best currently available evidence in a Markov decision analysis model to provide an important interim source of information [13,14] to inform the ongoing debate regarding best treatment for potentially resectable pancreatic cancer. On an intention-to-treat basis conclusive superiority of either pathway could not be concluded. However, Markov cohort analysis demonstrated superiority of NAT pathway if multimodal treatment was received. This highlights three important future directions for research: 1) cost-effectiveness analysis of NAT *versus* SF pathways 2) assessment of treatment pathways of resectable only cases hence offering a true like-for-like comparison and 3) developing methods of personalized predictive statistical modeling to provide individualized predictions of outcomes across competing treatment pathways to support shared clinical decision-making.

## Supporting information

S1 Fig PRISMA flowchart for phase II/II NAT trials. (TIF)

S2 Fig PRISMA flowchart for RCT trials of SF approach. (TIF)

S3 Fig PRISMA flow chart for non-RCT trials used in SF arm of the Markov model. (TIF)

S4 Fig Assessment of the risk of bias of all RCTs included in the SF arm of the Markov model. (TIF)

S1 Table Assessment of the risk of bias of all included studies in the NAT arm of the Markov model. (DOCX)

S2 Table Assessment of the risk of bias of all non-RCTs included in the SF arm of the Markov model. (DOCX)

S1 File PRISMA checklist. (PDF)
