# Supplementary figures and images for: Neoadjuvant therapy versus upfront surgery for potentially resectable pancreatic cancer: A Markov decision analysis
Source: PLoS One. 2019 Feb 28;14(2):e0212805. doi: 10.1371/journal.pone.0212805 (PMC6394923; doi:10.1371/journal.pone.0212805)

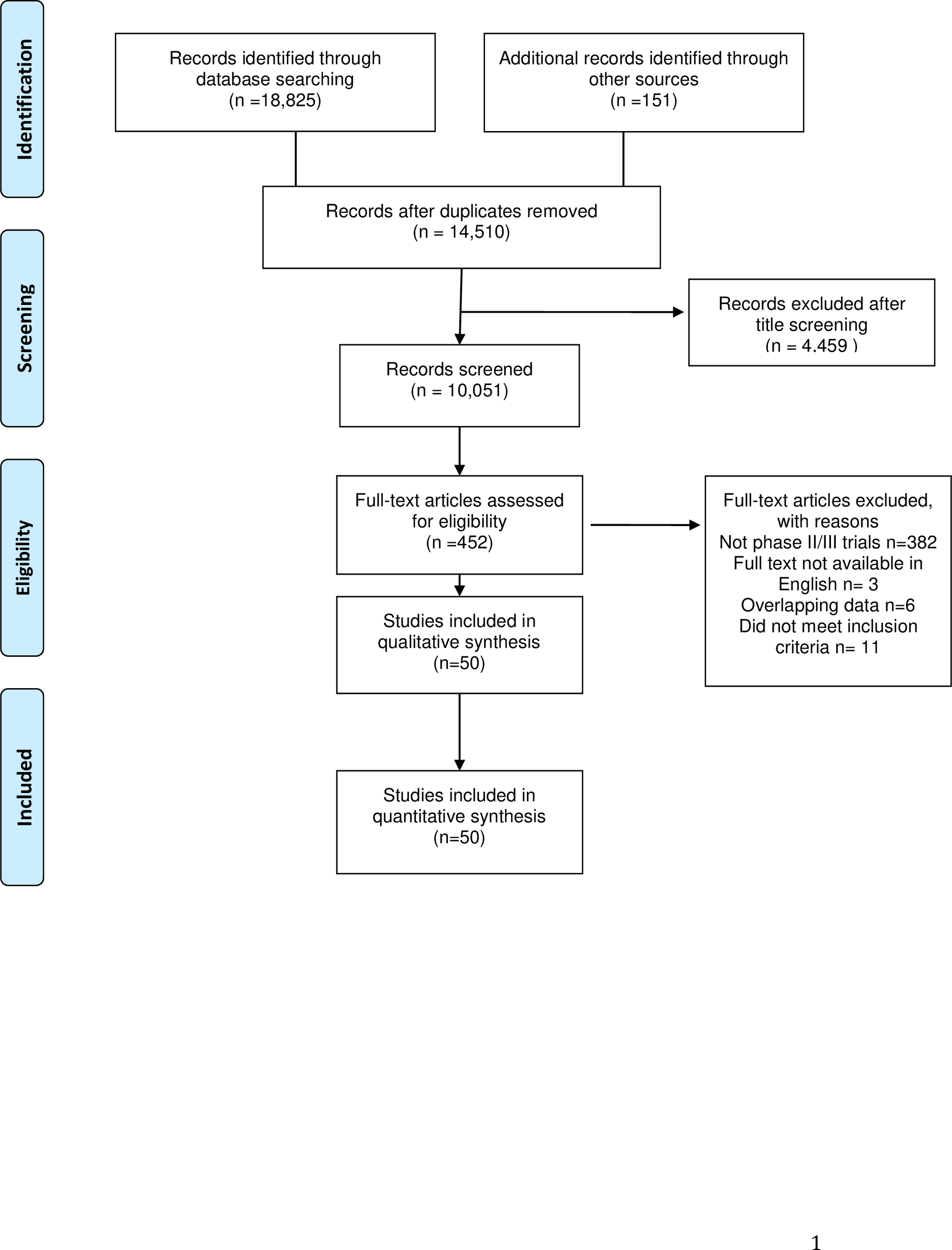

Supplement: S1 Fig — (TIF) [file pone.0212805.s001.tif]

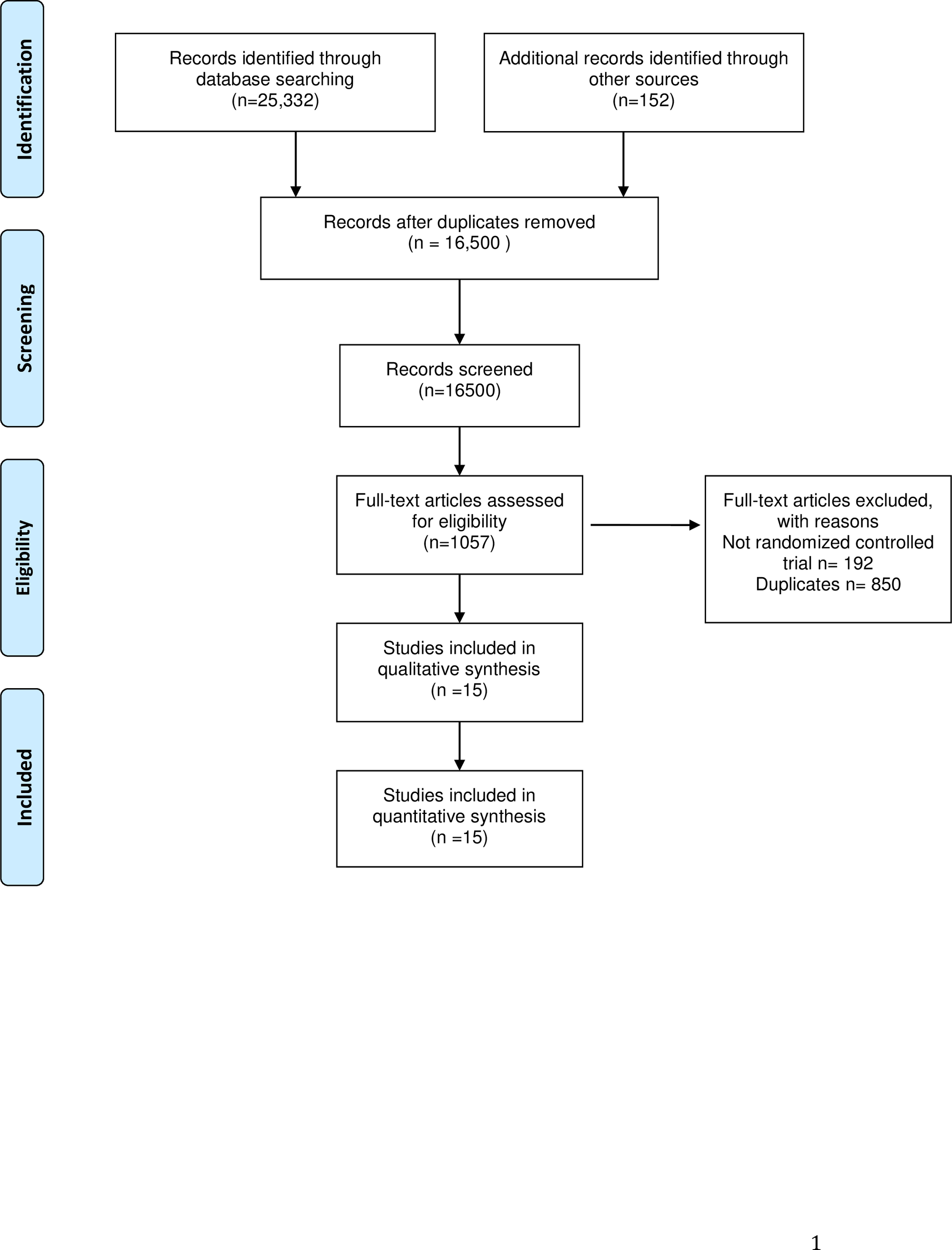

Supplement: S2 Fig — (TIF) [file pone.0212805.s002.tif]

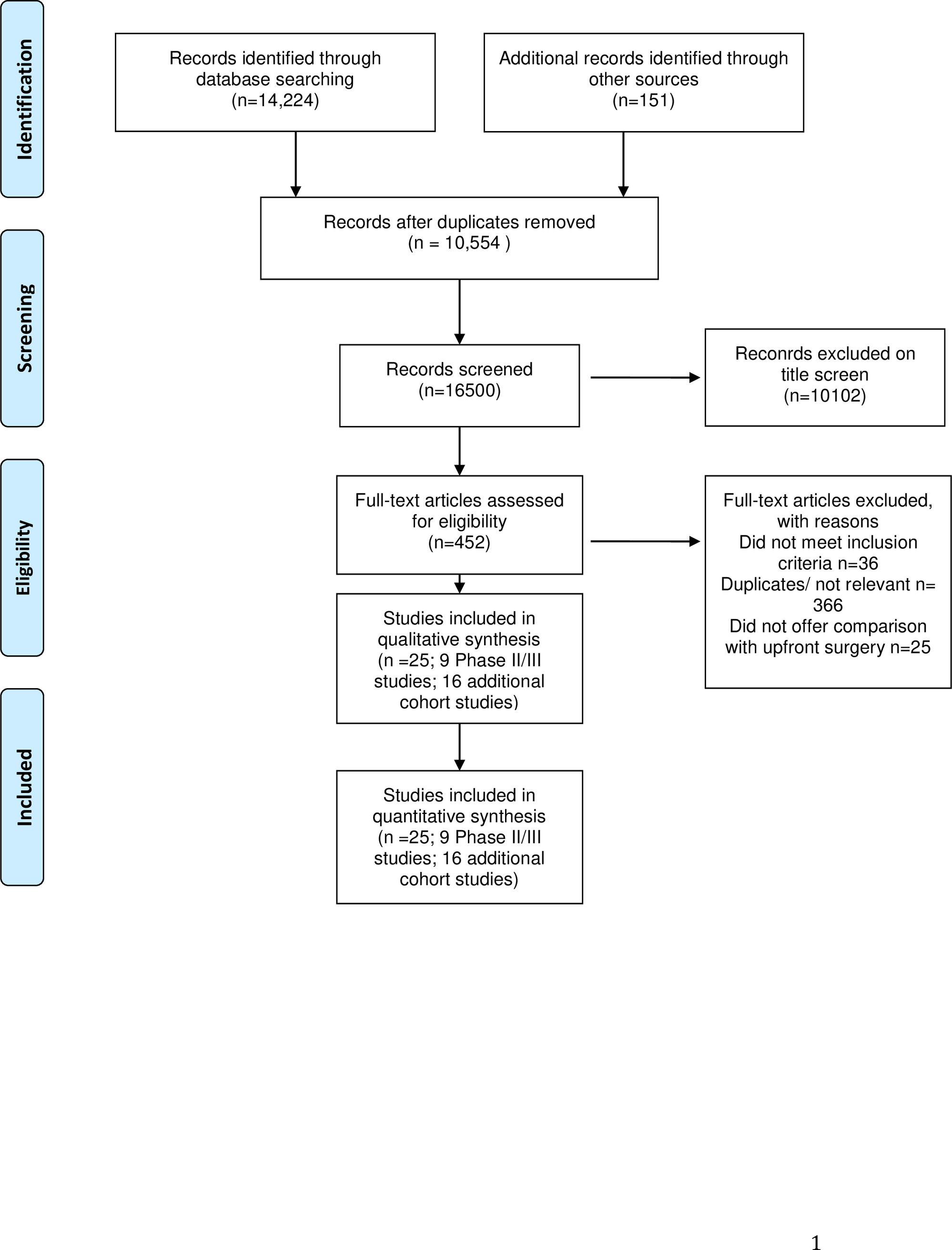

Supplement: S3 Fig — (TIF) [file pone.0212805.s003.tif]

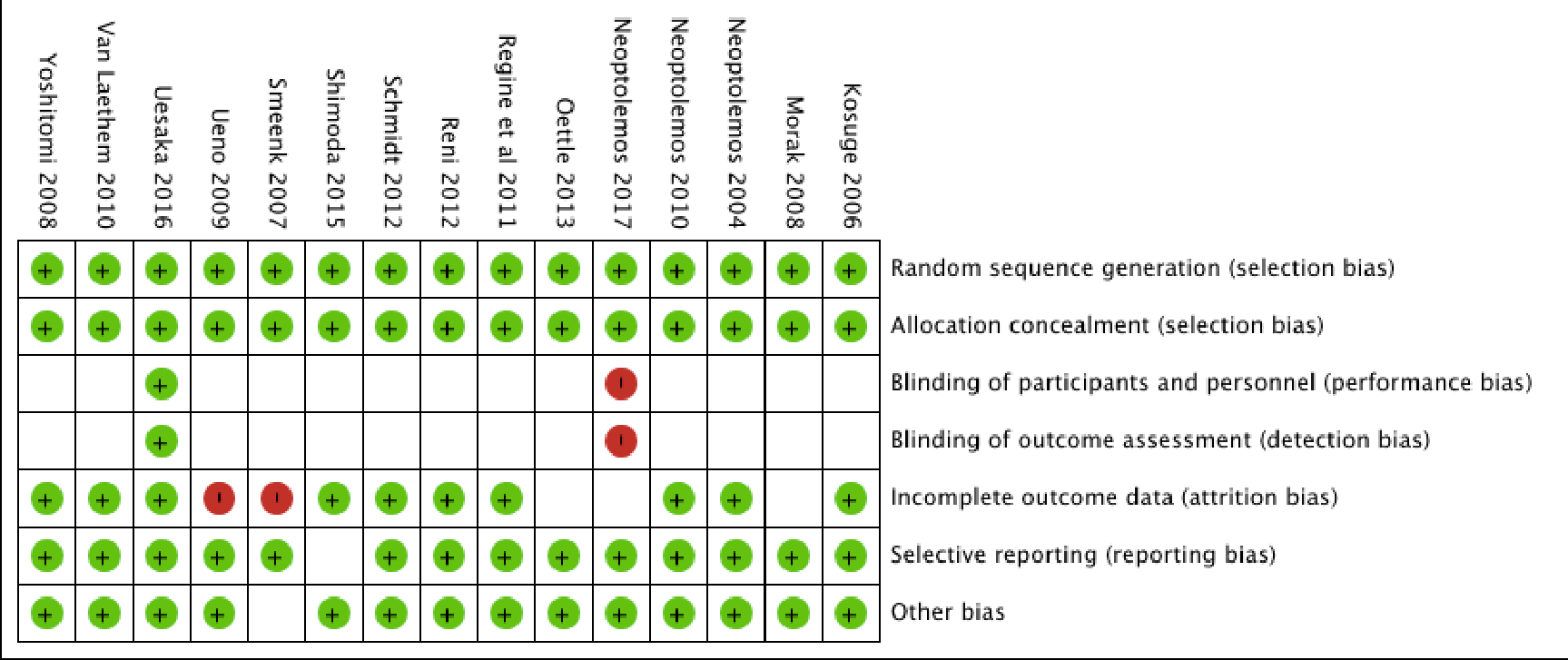

Supplement: S4 Fig — (TIF) [file pone.0212805.s004.tif]
