# Supplementary material for: Neoadjuvant therapy versus upfront surgery for potentially resectable pancreatic cancer: A Markov decision analysis
Source: PLoS One. 2019 Feb 28;14(2):e0212805. doi: 10.1371/journal.pone.0212805 (PMC6394923; doi:10.1371/journal.pone.0212805)
Supplement: S1 Table — (DOCX) [file pone.0212805.s005.docx]

| **Reference** | **No.** | **Single or Multicentre trial** | **Randomization** | **ROBINS-I risk of bias assessment** |
| --- | --- | --- | --- | --- |
| Al-Sukhun et al. [26] | 20 | Single | No | Moderate |
| Cardenes et al. [27] | 28 | Single | No | Moderate |
| Casadei et al. [28] | 18 | Single | Yes | Low/Moderate |
| Cetin et al. [29] | 11 | Single | No | Moderate |
| Chakraborty et al. [30] | 13 | Single | No | Moderate |
| Crane et al. [31] | 69 | Single | No | Low/Moderate |
| Epelbaum et al. [32] | 20 | Single | No | Moderate |
| Esnaola et al. [33] | 37 | Single | No | Moderate |
| Evans et al. [34] | 86 | Single | No | Moderate |
| Fiore et al. [35] | 34 | Single | No | Moderate |
| Golcher et al. [37] | 33 | Multicentre | Yes | Low |
| Golcher et al.[36] | 121 | Single | No | Moderate |
| Heinrich et al. [38] | 28 | Single | No | Moderate |
| Herman et al. [39] | 49 | Multicentre | No | Low/Moderate |
| Hong et al. [40] | 50 | Multicentre | No | Moderate |
| Jang et al.[41] | 27 | Multicentre | Yes | Low |
| Jensen et al. [42] | 23 | Single | No | Moderate |
| Joensuu et al. [43] | 33 | Single | No | Moderate |
| Kim et al. [44] | 68 | Multicentre | No | Low/Moderate |
| Landry et al. [45] | 21 | Multicentre | Yes | Low |
| Laurent et al. [46] | 22 | Single | No | Moderate |
| LeScodan et al. [47] | 41 | Multicentre | No | Low/Moderate |
| Lee et al. [48] | 43 | Single | No | Moderate |
| Leone et al. [49] | 39 | Single | No | Moderate |
| Lin et al. [50] | 42 | Single | No | Moderate |
| Lind et al. [51] | 17 | Single | No | Moderate |
| Magnin et al. [52] | 32 | Single | No | Moderate |
| Magnino et al. [53] | 23 | Single | No | Moderate |
| Marti et al. [54] | 26 | Single | No | Moderate |
| Mattiucci et al. [55] | 40 | Single | No | Moderate |
| Massucco et al. [56] | 28 | Single | No | Moderate |
| Maximous et al. [57] | 25 | Single | No | Moderate |
| Mornex et al. [58] | 41 | Multicentre | No | Low/Moderate |
| Motoi et al. [59] | 35 | Multicentre | No | Low/Moderate |
| Moutardier et al. [60] | 19 | Single | No | Moderate |
| O’Reilly et al. [61] | 38 | Single | No | Moderate |
| Palmer et al. [62] | 50 | Single | Yes | Low |
| \| Pipas et al. [63] \| \| --- \| | 37 | Single | No | Moderate |
| Pister et al. [64] | 37 | Single | No | Moderate |
| Sahora et al. [65] | 25 | Single | No | Moderate |
| Satoi et al. [66] | 35 | Single | No | Moderate |
| Sherman et al. [67] | 45 | Single | No | Moderate |
| Small et al. [68] | 29 | Single | No | Moderate |
| Talamonti et al. [69] | 20 | Multicentre | No | Moderate |
| Tinchon et al. [70] | 12 | Single | No | Moderate |
| Turrini et al. [71] | 34 | Multicentre | No | Moderate |
| Van Buren et al. [72] | 59 | Single | No | Moderate |
| Varadhachary et al. [73] | 90 | Single | No | Moderate |
| Vento et al. [74] | 22 | Single | No | Moderate |
| Wilkowski et al. [75] | 93 | Multicentre | Yes | Low |
