# Supplementary material for: Neoadjuvant therapy versus upfront surgery for potentially resectable pancreatic cancer: A Markov decision analysis
Source: PLoS One. 2019 Feb 28;14(2):e0212805. doi: 10.1371/journal.pone.0212805 (PMC6394923; doi:10.1371/journal.pone.0212805)
Supplement: S2 Table — (DOCX) [file pone.0212805.s006.docx]

| **Reference** | **Study Type** | **Multi or Single Centre** | **Randomization** | **No. SF cohort** | **ROBINS-I risk of bias assessment^21^** |
| --- | --- | --- | --- | --- | --- |
| Al-Sukhun et al. [26] | Phase II Trial | Single | No | 21 | Moderate |
| Casadei et al.[28] | Phase II Trial | Single | Yes | 20 | Moderate |
| Golcher et al. [36] | Phase II Trial | Single | No | 58 | Moderate |
| Golcher et al. [37] | Phase II Trial | Multiple | Yes | 33 | Low |
| Lind et al. [51] | Phase II Trial | Single | No | 35 | Moderate |
| Massucco et al. [56] | Phase II Trial | Single | No | 44 | Moderate |
| Satoi et al. [66] | Phase II Trial | Single | No | 41 | Moderate |
| Vento et al. [74] | Phase II Trial | Single | No | 25 | Moderate |
| Jang et al. [41] | Phase II/III Trial | Multiple | Yes | 23 | Low |
| DeGus et al. [91] | Retrospective | Multicentre | No | 6840 | Moderate/Serious |
| Mellon et al. [92] | Retrospective | Single centre | No | 241 | Moderate/Serious |
| Nurmi et al. [93] | Retrospective | Single centre | No | 150 | Serious |
| Shubert et al. [94] | Retrospective | Multicentre | No | 216 | Moderate/Serious |
| Artinya et al. [95] | Retrospective | Multicentre | No | 419 | Serious |
| Ielpo et al. [96] | Prospective | Single centre | No | 36 | Serious |
| Roland et al. [97] | Prospective | Single centre | No | 85 | Moderate/Serious |
| DeGus et al. [98] | Retrospective | Multicentre | No | 11316 | Moderate/Serious |
| Mokdad et al. [99] | Retrospective | Multicentre | No | 6015 | Moderate/Serious |
| Chen et al. [100] | Retrospective | Multicentre | No | 98 | Moderate/Serious |
| Tzeng et al. [101] | Prospective | Single centre | No | 52 | Moderate |
| Fujii et al. [102] | Prospective | Single centre | No | 71 | Moderate/Serious |
| Fujii et al. [103] | Prospective | Single centre | No | 416 | Moderate/Serious |
| Papalezova et al. [104] | Retrospective | Single centre | No | 92 | Moderate/Serious |
| Hirono et al. [105] | Prospective | Single centre | No | 124 | Moderate/Serious |
| Murakami et al. [106] | Retrospective | Single centre | No | 25 | Serious |
